# High-Performance Fluorescence Molecular Tomography through Shape-Based Reconstruction Using Spherical Harmonics Parameterization

**DOI:** 10.1371/journal.pone.0094317

**Published:** 2014-04-14

**Authors:** Daifa Wang, Jin He, Huiting Qiao, Xiaolei Song, Yubo Fan, Deyu Li

**Affiliations:** 1 State Key Laboratory of Software Development Environment, Beihang University, Beijing, China,; 2 Key Laboratory for Biomechanics and Mechanobiology of Ministry of Education, School of Biological Science and Medical Engineering, Beihang University, Beijing, China; 3 The Russell H. Morgan Department of Radiology and Radiological Sciences, Division of MR Research, Johns Hopkins University School of Medicine, Baltimore, Maryland, United States of America; Glasgow University, United Kingdom

## Abstract

Fluorescence molecular tomography in the near-infrared region is becoming a powerful modality for mapping the three-dimensional quantitative distributions of fluorochromes in live small animals. However, wider application of fluorescence molecular tomography still requires more accurate and stable reconstruction tools. We propose a shape-based reconstruction method that uses spherical harmonics parameterization, where fluorophores are assumed to be distributed as piecewise constants inside disjointed subdomains and the remaining background. The inverse problem is then formulated as a constrained nonlinear least-squares problem with respect to shape parameters, which decreases ill-posedness because of the significantly reduced number of unknowns. Since different shape parameters contribute differently to the boundary measurements, a two-step and modified block coordinate descent optimization algorithm is introduced to stabilize the reconstruction. We first evaluated our method using numerical simulations under various conditions for the noise level and fluorescent background; it showed significant superiority over conventional voxel-based methods in terms of the spatial resolution, reconstruction accuracy with regard to the morphology and intensity, and robustness against the initial estimated distribution. In our phantom experiment, our method again showed better spatial resolution and more accurate intensity reconstruction. Finally, the results of an *in vivo* experiment demonstrated its applicability to the imaging of mice.

## Introduction

Near-infrared fluorescence molecular tomography (FMT) is used for the three-dimensional (3D) localization and quantification of fluorescent targets deep inside turbid tissue. As a convenient and cost-effective small animal imaging modality, it can provide accurate visualization and quantification of the distribution of fluorescent tracers. Various applications have been proposed or carried out using this tool to monitor diseases at the molecular level, such as enzyme activity [1], mapping expressions of cancer markers [2], [3], and monitoring targeted drug delivery [4]. Davis *et al.* recently presented multicolor imaging to monitor two cancer markers simultaneously [5]. Although some devices for FMT are commercially available, the need for higher spatial resolution and more quantitative and reliable reconstruction hinders the wider application of this technique.

The recovery of 3D fluorescence distribution from boundary measurements is a nonlinear inverse problem. Because of the scattered light propagation inside turbid tissue media, the problem is highly ill posed and thus susceptible to data noise and model errors. The ill-posedness makes FMT reconstruction a significant challenge. As a solution, additional prior information is generally applied through different regularization techniques. Smooth distribution constraints are typically imposed through methods such as Tikhonov regularization [6]. Information on the sparse distribution is utilized through different compressed sensing techniques [7], [8]. Edge enhancement priors are utilized by penalizing the fluorescence intensity gradient as a regularized term, such as in the total variation method [9], [10], [11], [12]. The development of multimodality FMT systems [13], [14] has boosted the fusion of information derived from anatomical structures [15], [16]. High-density sampling [17], which increases the amount of boundary measurements, has also proven effective, and several studies have focused on investigating the optimal source–detector configurations for different kinds of FMT imaging systems [18], [19]. Although these advances have been critical to moving FMT from the laboratory to commercial applications, great challenges remain in order to obtain 3D fluorescence distributions stably and accurately.

In many specific applications, the distribution of fluorescent targets can be well described as the sum of a small number of subdomains (shapes) with constant piecewise intensities. This approximation is very suitable for tumor applications, where the fluorescence agent binds specifically to tumor tissue. With shape parameterization, the number of unknowns is greatly reduced, which in turn decreases the ill-posedness of the reconstruction. Shape parameterization has been applied to diffuse optical tomography [20], [21], bioluminescence tomography [22], and electrical impedance tomography [23]. For FMT reconstruction, several studies have used a piecewise constant assumption. Álvarez *et al.* [24] applied a level set to time-resolved FMT to implicitly impose shape constraints, where the distributions are recovered with the piecewise constant assumption and the lifetime is estimated using a gradient method. They performed a series of numerical simulations to verify its effectiveness. Despite the reduced ill-posedness, shape-based reconstruction is still a nonlinear and ill-posed problem, and the initial conditions critically affect its solution. To overcome this limitation, Laurain *et al.* [25] extended topological sensitivity analysis to generate good initial estimates for shape-based FMT and evaluated the effectiveness through numerical simulations. In our previous work, we performed shape based reconstruction by assuming the fluorescent targets to be regular ellipsoids [26]. A two-step solver was developed to enhance the robustness against the initial values and noise, and graphics processing unit (GPU) acceleration was adopted to accelerate the computation of the Jacobian matrix and gradient.

In this work, we developed a novel shape-based reconstruction method by introducing spherical harmonics [27] for shape modeling. Compared to our previous ellipsoid approximation, spherical harmonics can better model irregular targets [20], [23], [28], which leads to more accurate recovered images. In the proposed method, the inverse problem is parameterized with respect to the spherical harmonics coefficients of the shape boundaries. To stabilize the solution, the two-step strategy is expanded, and a modified block coordinate descent approach is introduced to recover shape parameters. Since the computation of the Jacobian matrix and gradient with respect to the spherical harmonics coefficients is rather complex and time-consuming, we accelerate their calculations by using GPU based on our previous work [26] on ellipsoid shape parameters. We evaluated the proposed method using numerical simulation, a physical phantom, and *in vivo* data, and it demonstrated much better performance than conventional voxel-based reconstruction.

## Methods

### Forward problem

In turbid tissue media, the light propagation for source–detector separations of more than several millimeters can be modeled by a partial differential equation called the diffusion equation. By setting the spatially localized impulse function 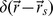 as the source term, the Green's function 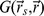 can then be solved via numerical techniques such as the finite element method (FEM) [29], [30]. By considering that light travels from 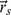 to position 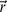 and from 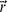 to detector 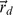 and integrating over the whole imaged domain 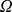, the forward mapping from the fluorescence distribution 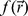 to the received fluorescence signal 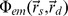 for the source–detector pair 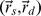 can be expressed as
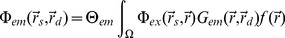
(1)where 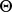 represents the total system amplification factor from the quantum efficiency, detection efficiency, etc. The subscripts 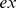 and 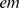 indicate the excitation light and emitted fluorescence wavelengths, respectively. In FMT reconstruction, using the normalized Born ratio 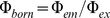 of the corresponding measurements at the emission and excitation wavelengths has been proven to provide much more robust performance with respect to the uneven system amplification factor 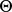 and unknown heterogeneity of the imaged medium compared to using the fluorescence signals alone [31]. Given Green's functions, the normalized Born ratio can be written as follows:



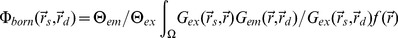
(2)For numerical computation, the above integral equation is generally discretized using the piecewise constant voxel basis as follows:
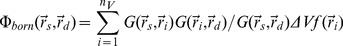
(3)where the imaged domain is divided into 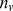 uniform voxels with volume 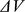. For data from all 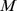 source–detector pairs, a matrix-vector product form can be generated from Eq. (3):
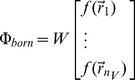
(4)where 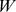 is the weight matrix with size 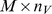. The above linear system is highly ill posed, which makes direct inversion impossible. A priori information is typically required for stabilization, such as smooth constraints imposed via Tikhonov regularization.

We assumed that the fluorescent targets have sharp interfaces and are distributed as piecewise constants. That is, the imaged domain 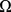 can be split into 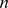 disjointed subdomains 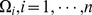 and the remaining background 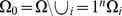 with constant concentrations 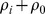 and 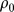, respectively. Then, the fluorescence distribution is expressed as follows:
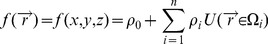
(5)where 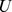 is the unit step function. In our previous work [23], we used ellipsoids to approximate the subdomains for simplicity; however, this approach is limited with regard to modeling irregular geometries. Spherical harmonics can represent fairly intricate 3D polar shapes well (a polar shape can be described as a single-value function in spherical coordinates with respect to a center position). Since more accurate shape modeling yields better shape reconstruction performance, we adopted real-value spherical harmonics to parameterize the arbitrary 3D subdomain boundaries 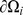, as introduced in [28]. Then, the surface locations 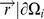 of boundary 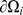 are represented in spherical coordinates with respect to a given center 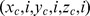:
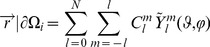
(6)where 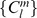 are the expansion coefficients and 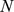 is the maximum degree of spherical harmonics used. The real value basis function 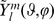 is defined as follows:
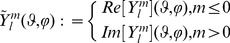
(7)where 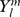 are the spherical harmonics functions of complex values [24]. Then, a single fluorescence inclusion can be parameterized using 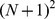 expansion coefficients for up to 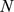-order spherical harmonics, the center position, and the fluorescence concentration. In addition to the background concentration 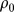, a total of 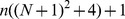 shape parameters model the piecewise constant fluorescence distribution with 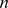 disjointed subdomains, which can be depicted by the new notation 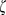. In this study, we used second-order spherical harmonic coefficients.

### Inverse problem

To recover the shape parameters 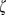, a least-squares minimization function is established to minimize the difference between theoretical predictions and practical measurements:
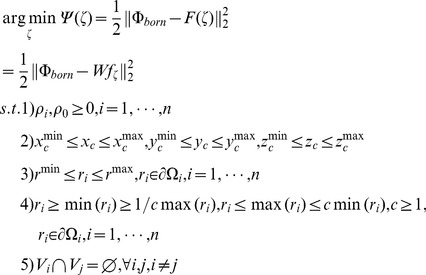
(8)Herein, given a previously defined uniform voxel discretization with sufficient small size such as 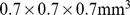, the theoretical predictions 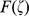 are computed via a matrix-vector product based on Eq. (4). The fluorescence distribution vector 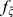 is generated by transforming the shape parameters to the voxel grids through Eqs. (5)–(7). For stable reconstruction, reasonable constraints are imposed to the shape parameters. The first constraint is the nonnegativity of the fluorescence intensity. The second is to restrict the shape centers inside box bound of the image object. The following two constraints prevent the radius from being too small or large and geometric shapes from being too narrow. The final constraint (non-overlap) guarantees the disjointedness of the subdomains.

**Figure 1:**
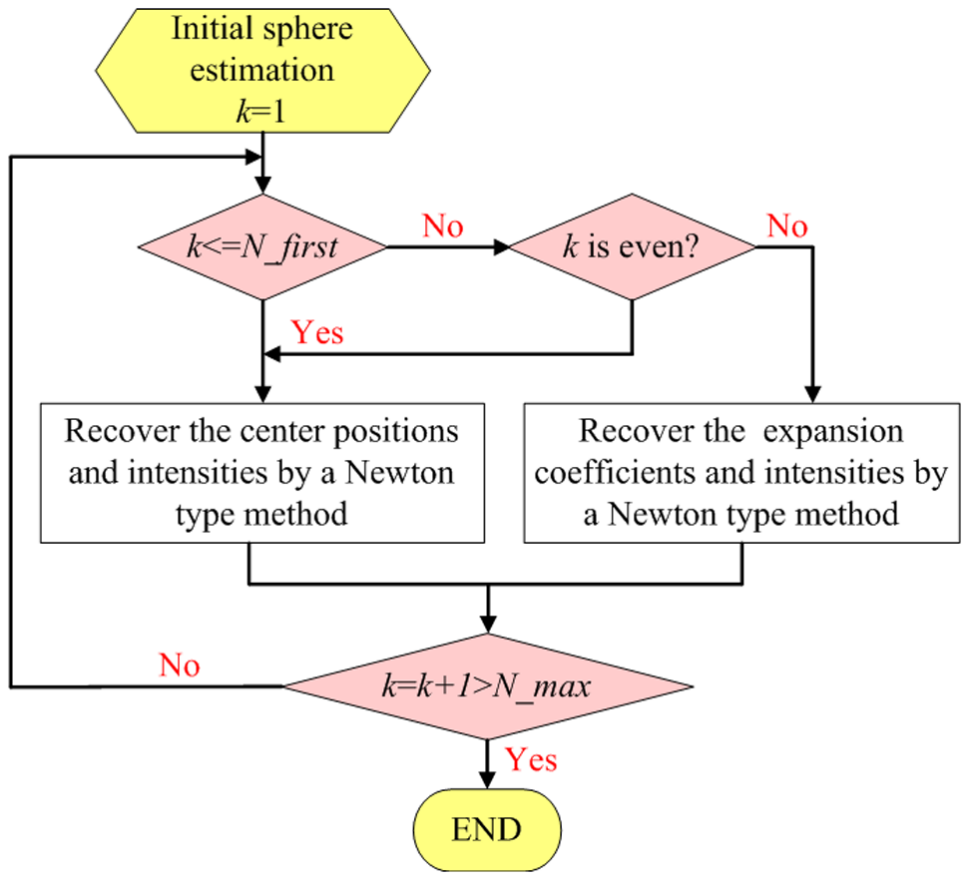
Optimization of spherical harmonics shape parameters. In the first step (the initial *N_first* iteration), the unknown targets are assumed to be spheres. In the second step (the following iterations until the maximum iteration number *N_max*), a modified block coordinate descent strategy is adopted to alternately recover the grouped shape parameters.

Although the unknowns are greatly reduced because of the spherical harmonics, Eq. (8) is still a complex nonlinear problem. An appropriate iterative solver is needed for its numerical solution, which relies on the shape gradient and Hessian matrix. However, gradient-based solvers are extremely sensitive to the initial conditions, and getting a good initial estimate inside the imaged object is generally a difficult and challenging task. In our previous work [26], since different types of shape parameters contribute differently to the measurement data, we handled the ellipsoid shape parameters using a two-step strategy and proved its capability of improving the robustness against initial conditions. Based on the previous work, we introduced a two-step and modified block coordinate descent strategy for spherical harmonics–based shape reconstruction, as shown in Fig. 1. In the first step, by setting the initial shapes as spheres, Eq. (8) is solved with the spherical harmonics expansion coefficients as invariants; this yields relatively good initial conditions for the next step, especially for the center positions. A modified block coordinate descent strategy is then employed in the second step. That is, the parameters of blocks 1 (center positions and intensities) and 2 (spherical harmonics expansion coefficients and intensities) are separately estimated at even and odd iterations. Herein, the center positions and spherical harmonics expansion coefficients are placed into different blocks, as they have weak logical connections and can be separated. However, the fluorescence intensities are put into both blocks since they have strong logical connections with the other shape parameters. This is different from the standard block coordinate descent method, where each variable appears in only one block. As shown in Fig. 1, the maximum iteration number of each step (*N_first* and *N_max* minus *N_first*) obviously influences the final reconstruction result. Empirically, *N_first* was set to 20. This number is sufficient to get a good estimation of the center positions; a larger value does not produce an obvious improvement but requires more computation time. For the second step, more iterations generally yield a better shape but may produce over-optimization. This is because of the high ill-posedness of the recovered block 2; details are discussed later in the discussions and conclusion section. In our experience, *N_max* can be set to a relatively low value such as 40 in the presence of a high level of noise and model error. *N_max* can be set to a relatively large value such as 80 in the presence of a moderate level of noise and model error.

In each step, a Newton-type method is used as an iterative solver for the nonlinear minimization problem, where the update 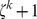 for 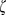 is given by
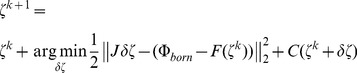
(9)Herein, 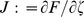 is the Jacobian matrix. 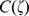 is the penalty term because of the shape constraints 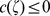 and is imposed through the popular exterior penalty function method. Then, a minimization program with an increasing sequence of penalty parameters 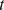 as 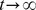 is generated:
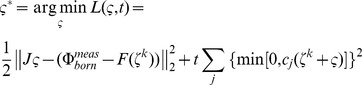
(10)where 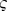 is a new notation to denote 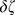. In each t-sub problem, 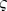 is updated via Newton's method, and 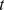 is doubled:
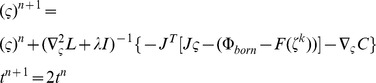
(11)where the Hessian matrix 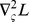 is 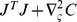. Since 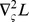 is poorly conditioned, regularization is added with parameter 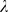 for stable inversion, and an iterative solver is used with the symmetric LQ method (MATLAB function symmlq). The parameter 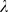 was empirically selected to be 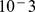 and worked well in the simulation and physical experiments.

Generally, the background volume is much larger than the targets. To improve the conditioning of the inverse problem, the background fluorescence intensity is scaled during reconstruction:
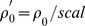
(12)In this study, the scale factor 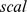 was set to 100.

### Computation of objective function value, gradient, and Jacobian matrix

For Eq. (8), the Jacobian matrix computation can be expressed as
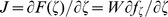
(13)


Since 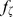 is a nonlinear function of 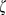, the perturbation method is used to compute 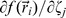 using a sufficient small perturbation 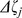:
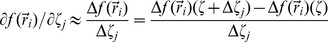
(14)Eqs. (8), (13), and (14) show that the shape-voxel mapping 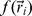 is the basic component for evaluating 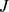 and 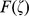 and that it is critical to evaluating the non-overlap constraint. As no analytical expression is available for this nonlinear mapping, we can directly calculate 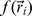 by uniformly dividing the corresponding voxel into 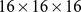 fine sub-voxels with centers 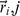:
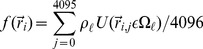
(15)Herein, the point-in-shape test 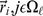 is performed according to Eqs. (6) and (7).

**Figure 2:**
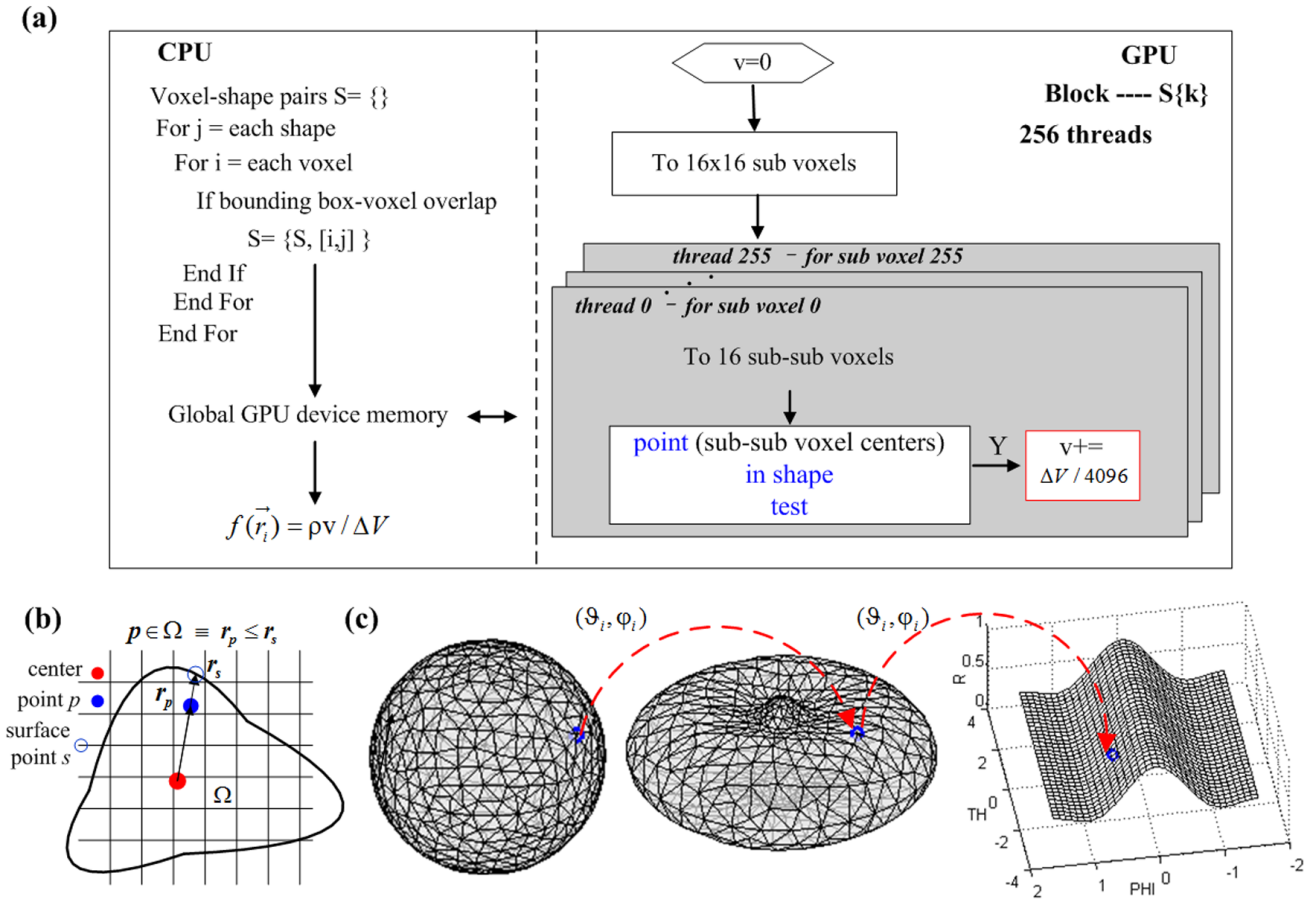
Shape-voxel mapping. (a) Flowchart of the GPU-accelerated computation of shape-voxel mapping. (b) Illustration of how to determine whether a point is inside a polar shape. (c) Digitalization of spherical harmonics parameterized shape with triangular mesh surface. The parameterized-shape surface mesh (middle) is generated by mapping a predefined triangular meshed unit sphere surface (left). For each mesh node (e.g., blue circle), its new radius on the parameterized-shape surface is found by keeping its 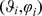 unchanged. Further, a regular table is generated in the spherical coordinates 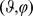 space from these mesh nodes for faster point-in-shape determination.

Similar to our previous work [26], the frequently performed basic operations (i.e., weight matrix multiplication and shape-voxel mapping) take more than 90% of the computation time. Hence, we accelerated them using the advanced CUDA GPU platform [32], [33], where the former is performed using the standard CUDA CUBLAS library and the latter is performed as shown in Fig. 2(a). Generally, a shape target is small compared to the whole imaging domain, and processing the many non-overlapped voxel-shape pairs using GPU is inefficient [32], [33]. Hence, a voxel-shape paring procedure is first performed with CPU by judging the overlap between a voxel and the bounding box of a shape. GPU is then used to determine the concrete overlapped volume for each voxel–shape pair through concurrently executed threads.

As shown in Fig. 2(a), the point in shape test 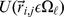 is the most important component of the shape-voxel mapping. For the polar shape, this test is performed by comparing the radius 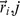 from the shape center to the point and the radius 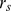 of the corresponding shape surface point, as illustrated in Fig. 2(b). Theoretically, 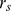 can be directly calculated using Eq. (6); however, this is time-consuming. For faster computation, we adopted an interpolation technique. A triangular-mesh unit sphere surface is introduced where each mesh node has a pair of spherical coordinates 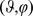, as shown in Fig. 2(c). These mesh nodes can then determine the parametric surface by finding their new distances from the center through Eq. (6). The unit sphere transformation is inspired by [28], where the mapped mesh was mainly used for boundary element method discretization and solution. The 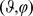 space is then uniformly refined to 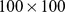 grids, and the corresponding radii are calculated by interpolation from those mesh nodes. Then, given a point 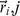 with spherical coordinates 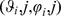, the radius of the corresponding surface point 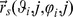 can be easily determined through two-dimensional interpolation among the four neighbor points in the 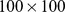 regular grids. As this lookup table operation is rather simple, it can be easily implemented through GPU.

### Voxel-based reconstruction

In the experiments, the proposed method was compared with traditional voxel-based reconstruction. In general, voxel-based reconstruction is formulated as a linear system:
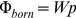
(16)where 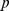 is the fluorescence distribution in 3D voxels and 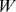 is the weight matrix as described in Eq. (4). The linear system is ill posed, which means that direct inversion is impossible. In this study, two techniques were used for its solution: the random access algebraic reconstruction technique (R-ART) with nonnegative constraints, which has been widely applied for FMT [34], [35]; and Tikhonov regularization, where Eq. (16) is transformed into an *L_2_* regularized solution:
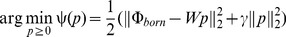
(17)where 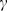 is the regularization parameter. The regularized least-squares problem is solved by using the conjugation gradient method, and the nonnegative constraints are imposed through the exterior penalty function method.

## Experiments and Results

The performance and effectiveness of the proposed method was evaluated through numerical simulations and experiments with a physical phantom and a mouse *in vivo*. All reconstructions were performed on our desktop computer, which has an Intel 2.8-GHz quad-core CPU, 16 GB RAM, and an NVIDA GTX 480 graphics card.

### Numerical simulations

A series of simulations was performed to compare the proposed method with the traditional voxel-based method and evaluate its performance in the presence of noise and background contrasts.

**Figure 3:**
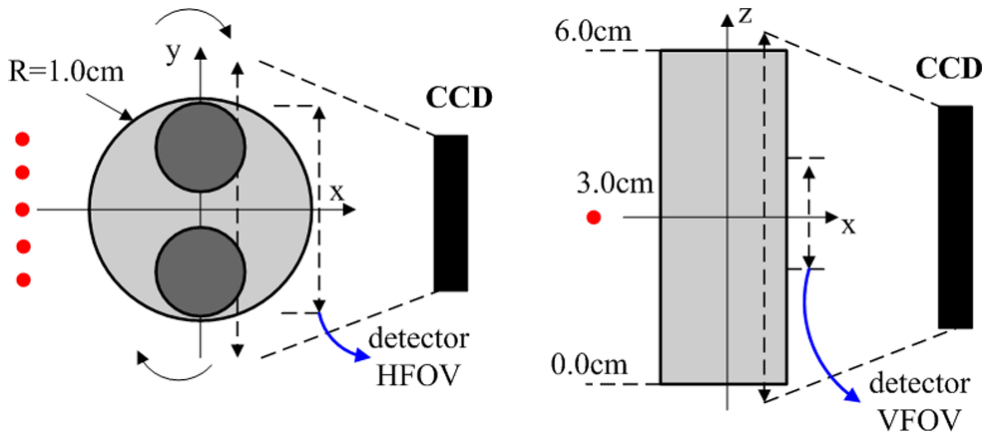
Simulation experiment sketch. A full-angle CCD camera–based imaging system configuration was used for the data simulation. The imaged object was a cylinder phantom with two embedded cylinder heterogeneities (different absorption coefficients). For each projection, five different excitation sources were scanned (red dots), and the detectors, which corresponded to selected detection points on the image plane, were within 1.8 cm of the detector horizontal FOV (HFOV) and 2.2 cm of the detector vertical FOV (VFOV) with a detector spacing of 0.2 cm.

To mimic the heterogeneous optical properties of a real mouse, a cylinder model (6.0 cm height and 2.0 cm diameter) with two cylindrical heterogeneities (6.0 cm height and 0.35 cm diameter) was used, as shown in Fig. 3. Reasonable optical properties were chosen with a background of 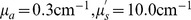 and heterogeneity of 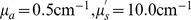. Full angle data-acquisition was adopted [34], where data were simulated for 24 evenly distributed projection angles around the model. For each projection angle, the light source was sequentially scanned over five positions in steps of 0.3 cm to generate five projections. For each projection, the detector sampling on the charge coupled device (CCD) detection field of view was over a 1.8 cm×2.2 cm region with 0.2 cm spacing. The data simulations were performed using FEM.

In the reconstructions, we simply assumed the imaged object to be homogenous with optical properties of 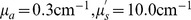 to mimic the unknown heterogeneity in practical cases. During all of the numerical experiments, the same geometry constraints were applied with 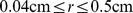, 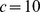, 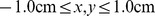, and 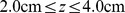. Eighty iterations (one update in Eq. 9 corresponds to one iteration) were performed in the shape-based reconstructions, where the first step took 20 iterations. The 3D voxels for the Jacobian matrix calculation were inside the cylinder model and over 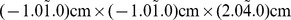 with a voxel size of 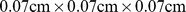. In the comparison experiments, 3D voxels were also used in voxel-based reconstruction. In the voxel-based reconstruction, both R-ART and Tikhonov regularization were adopted. R-ART was iterated 200 times with a relaxation parameter of 0.1. The Tikhonov regularization parameter was empirically set to 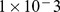, which gave a good balance between stability and smoothing. The conjugate gradient method was performed until the relative difference between neighboring iterations was less than 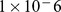.

#### Reconstruction of dual inclusions of different shapes

We evaluated the proposed method with closely placed dual inclusions of various shapes; these included ellipsoids, cuboids, and triangular prisms. The parameters are specified in Table 1. There was no fluorescence in the background. Then, 5% Gaussian noise was added to the synthetic measurements.

**Table 1:** Parameters for inclusions with various shapes.

ellipsoid	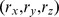	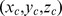	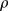
target 1	(0.22 0.11 0.15)	(0.00 0.25 3.00)	1.00
target 2	(0.22 0.11 0.15)	(0.00–0.25 3.00)	1.00

The second column lists the geometric dimensions: radii for the ellipsoid, edge length for the cuboid, and edge and height for the triangular prism.

**Figure 4:**
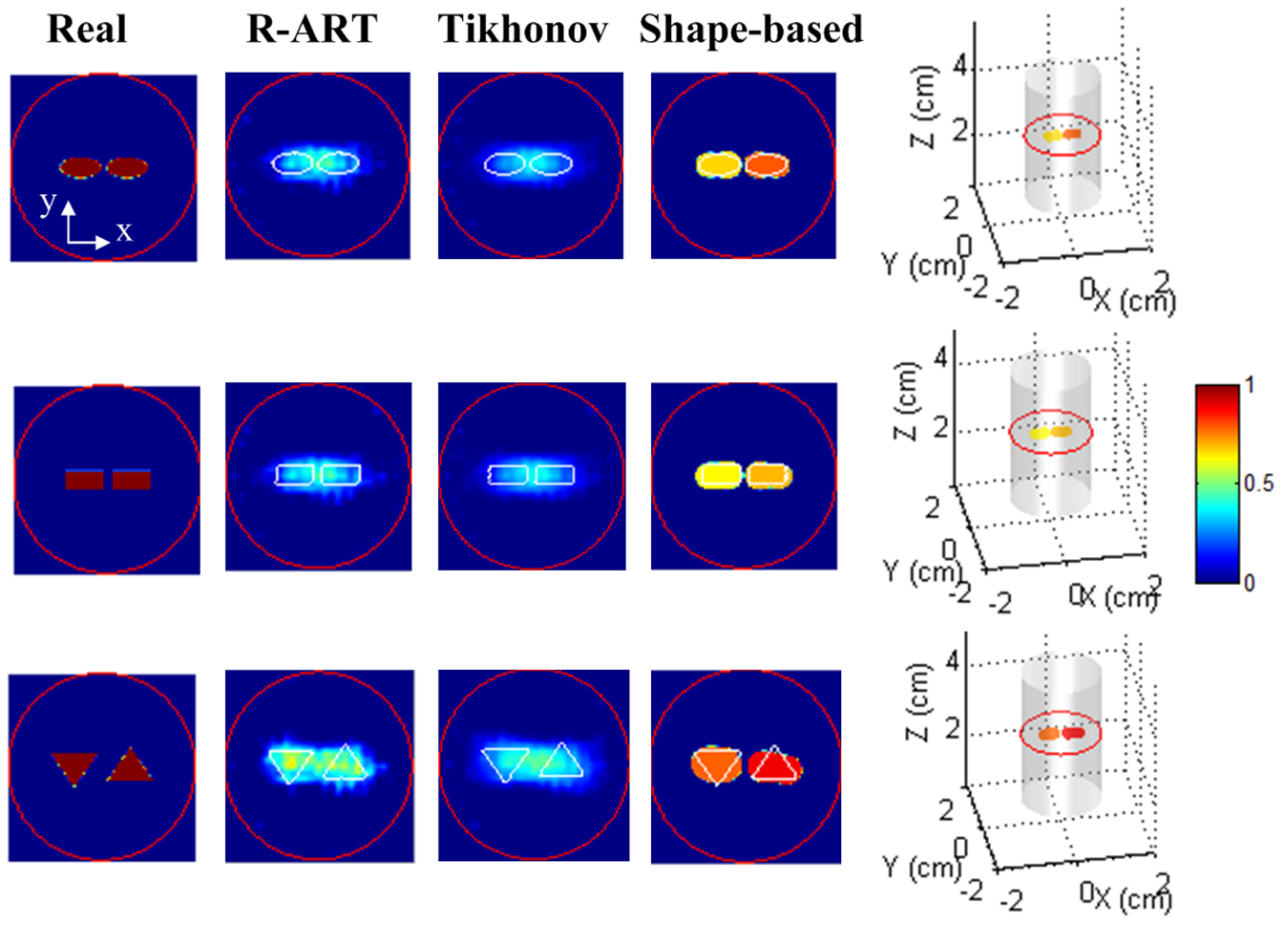
Comparison of the results from the proposed method and voxel-based reconstructions. In the slice images, the red circles denote the outer boundary of the imaged object, and the white lines denote the boundaries of the real inclusions. The slice images are of 3.0 cm height.

As shown in Fig. 4, neither R-ART nor Tikhonov regularization could resolve the dual targets for all of the different shapes. In contrast, because of the shape parameterization, the proposed method demonstrated much better resolution capability. It clearly separated the adjacent dual inclusions and matched intensities and morphology well. The free background was also accurately estimated. Of course, because of the high photon scattering in tissues and the presence of noise and heterogeneity, fully accurate recovery of the true shapes was still impossible. The better resolution capability of the proposed method is because of the successful utilization of shape priors, which greatly reduces the space of possible solutions. In other words, the shape-based method can find a better solution without getting stuck in the many cut solution branches.

As a benefit of the parallel acceleration by GPU, the shape reconstruction time was typically within several minutes. For example, the shape optimization for the dual ellipsoids took about 159 s. Without GPU, the computation time was about 83 min, which was 31 times longer.

**Figure 5:**
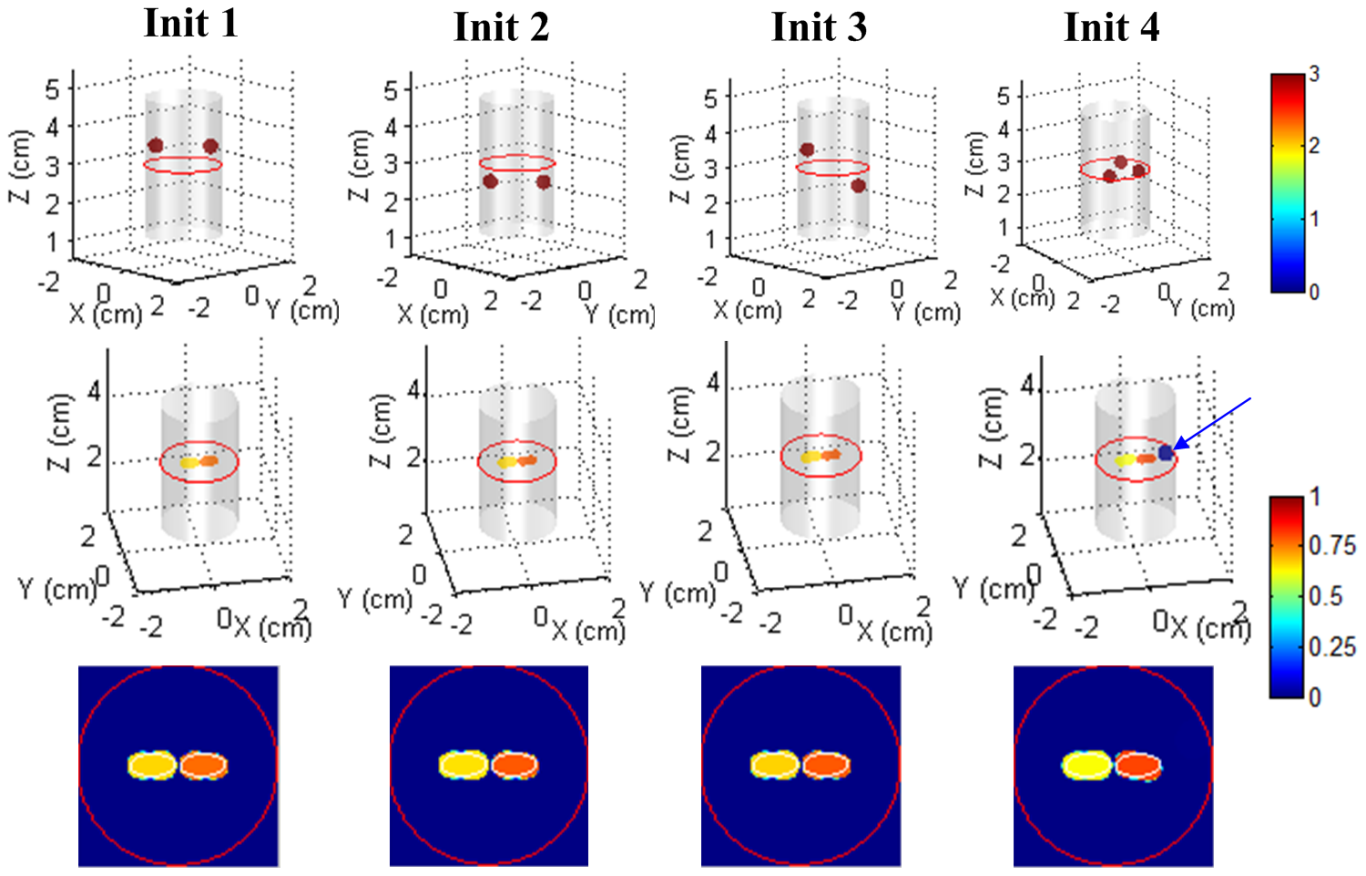
Reconstruction results with different initial estimates. In the slice images, the red circle denotes the boundary of the imaged object, and the white lines denote the boundaries of the real inclusions. The slice images are of 3.0 cm height.

To evaluate the performance with respect to different initial values, we selected dual spheres with different center distances away from the true inclusions, which represented initial shapes with different extents of goodness. As shown in Fig. 5, the proposed method worked well and demonstrated robust performance since it considered and handled the difference among shape parameters through the two-step and modified block coordinate descent strategy. In contrast, although not shown here, the straightforward method of simply recovering all parameters simultaneously generally corrupted the reconstruction process. The objective function value could not be decreased effectively, and the true inclusions were not found.

In some cases, it may be impossible to reliably determine the targets number *a priori*. By assuming more targets than actually needed, the proposed method can handle this problem to some extent. As demonstrated in Fig. 5, with three initial targets, the proposed method still recovered the true targets well, whereas the false target was reconstructed with ultra-low intensity.

#### Different noise level

**Figure 6:**
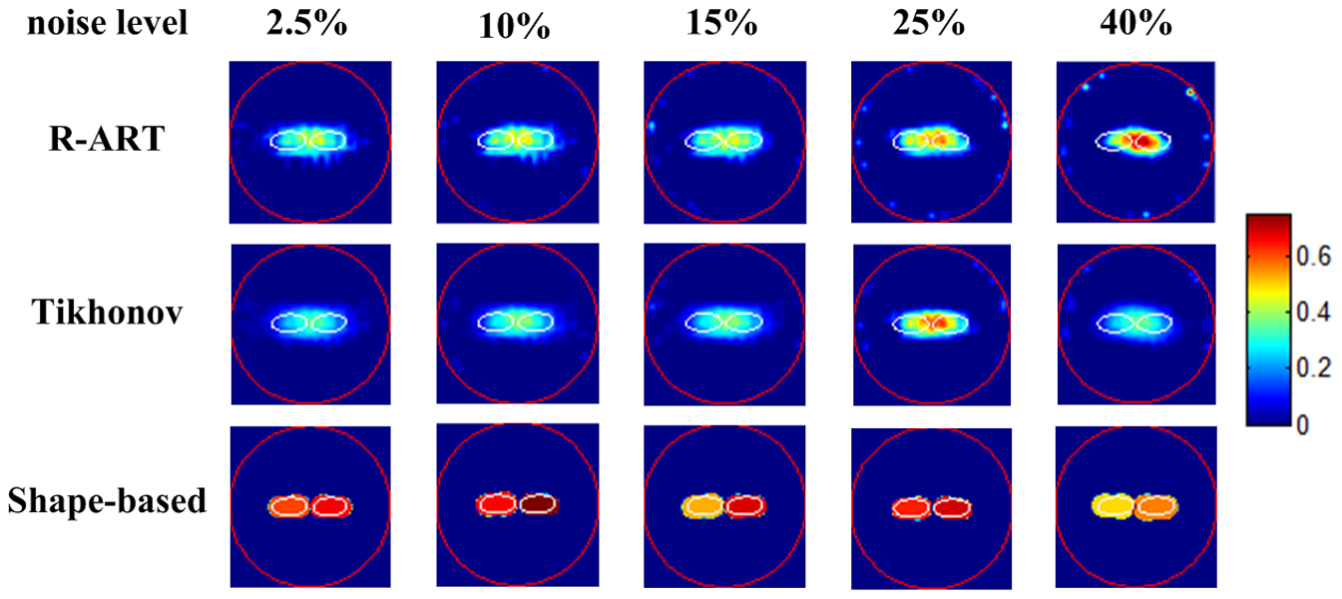
Reconstruction results of different noise levels. The red circle denotes the boundary of the imaged object, and the white lines denote the boundaries of the real inclusions. The slice images are of 3.0 cm height.

To evaluate the sensitivity of the proposed method to noise, different levels of Gaussian noise (2.5%–40%) were added to the synthetic measurements. The dual ellipsoids case was used as the configuration of the fluorescent targets, and the background was fluorescence-free. As shown in Fig. 6, neither voxel-based method could resolve the targets even at the lowest noise level. In contrast, the proposed method clearly separated and estimated the adjacent dual inclusions for various noise levels up to 40%; thus, it showed strong robustness against noise jamming.

#### Different background contrast level

Even state-of-the-art fluorescent probes still find it difficult to completely bind to targets without residuals in the background. Thus, we evaluated the performance of the proposed method using different contrast levels from 100∶1 to 10∶20. The dual ellipsoids case was used as the configuration of the fluorescent targets. In addition, 1% Gaussian noise was added to the synthetic measurements.

**Figure 7:**
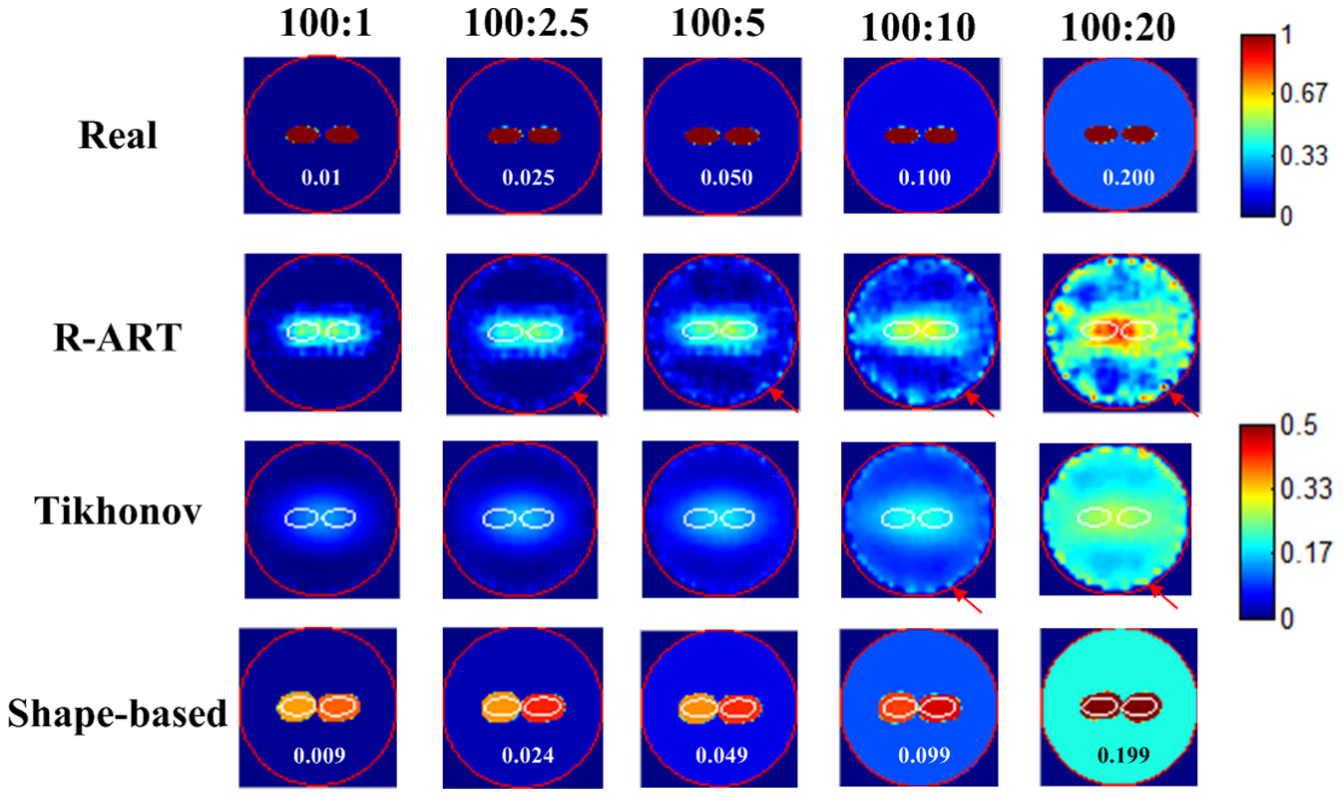
Reconstruction results of different fluorescence contrasts. The red circle denotes the boundary of the imaged object, and the white lines denote the boundaries of the real inclusions. The slice images are of 3.0 cm height.

As shown in Fig. 7, the background fluorescence could not be properly estimated by both voxel-based methods; both showed an obvious nonuniform distribution in the background region. In addition, the boundary artifacts gradually increased with the background fluorescence. For Tikhonov regularization, its spreading and smoothing effects became more evident in the presence of background fluorescence, especially at a low contrast level. Similar to the previous background-free case, the dual targets could not be resolved. In contrast, the proposed method demonstrated much better resolution capability and quantification. For all contrast levels, the dual targets were clearly separated. The background value was accurately reconstructed with a small absolute error that was within 0.001. These results verified the effectiveness of the proposed method under low fluorescence contrast conditions.

### Physical experiments

**Figure 8:**
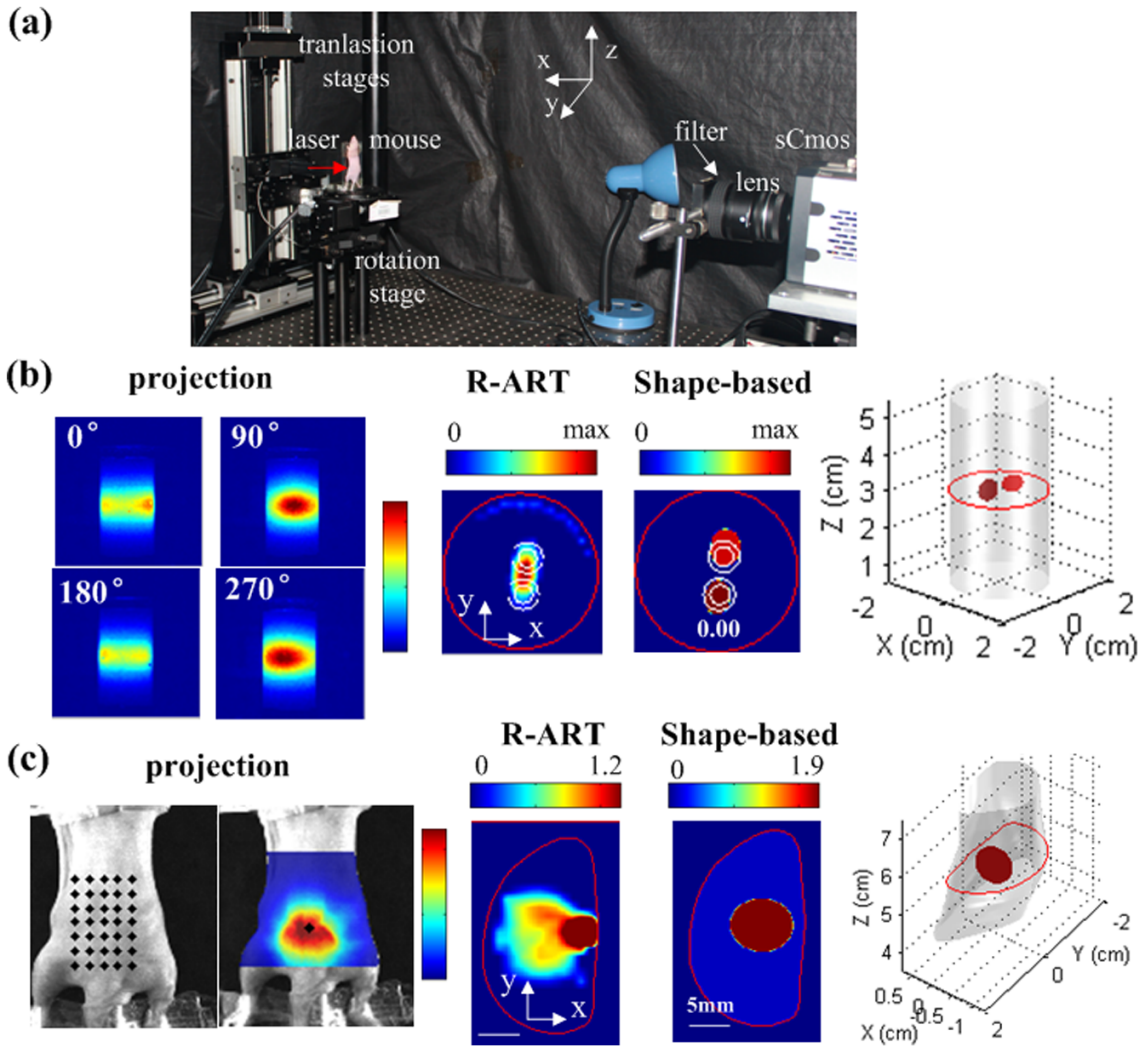
Physical experiments. (a) The full angle fluorescence molecular tomography system developed in-house. (b) Physical phantom experiment. Two fluorescence inclusions were placed closely together inside a cylinder phantom with a 0.10 cm edge-to-edge distance. The white circles denote the inner (solid line) and outer (dash line) boundaries of the real inclusions. (c) *In vivo* experiment. A fluorescence inclusion was embedded inside a nude mouse. The grid of black dots overlaid on the mouse represents the excitation light sources. For the slice images in (b) and (c), the red circle denotes the boundary of the imaged object, and every two slice images are at the same height, as depicted by the red circle in the corresponding 3D image.

Physical phantom and *in vivo* experiments were performed to evaluate the feasibility of the proposed method for practical applications. Our fluorescence imaging system, which was developed in-house, was used for data acquisition, as shown in Fig. 8(a). The imaged object was placed on a rotational stage for multiple angle image acquisition. The laser and detector were placed on opposite sides of the stage. The semiconductor laser (785 nm wavelength) output a small laser spot around 1 mm in diameter with a power of 14 mW. The detector was a highly sensitive sCMOS camera (Neo, Andor, Belfast, Northern Ireland, U.K.) coupled with a Nikkor 60 mm f/2.8D lens (Nikon, Melville, NY). The camera had a large chip area of 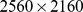 pixels with a 16-bit dynamic range. During the data acquisition, the sCMOS chip was cooled to 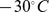 to reduce dark current noise. A neutral density filter of 1% transmittance (Daheng, Beijing, China) and 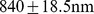
band-pass fluorescence filter (Semrock, Rochester, NY) were used for excitation and fluorescence image collection, respectively. In addition, 72 white light images were collected to reconstruct the object's 3D surface [36].

In the phantom experiment, a glass cylinder (inner diameter of 2.43 cm and outer diameter of 2.83 cm) was filled with intralipid (1% concentration 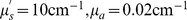). Two fluorescence inclusions were embedded closely together with a 0.10 cm edge-to-edge distance. Each inclusion was produced by pouring 40 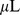 of indocyanine green (concentration of 4 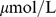) into a transparent glass tube (0.3 cm inner diameter and 0.5 cm outer diameter). Excitation and fluorescence data were collected at 36 projection angles evenly distributed over 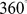. For each projection angle, three excitation positions along the horizontal direction were scanned sequentially at a distance of 0.3 cm. For voxel-based reconstruction, 50 R-ART iterations were performed with a relaxation parameter of 0.05. For shape-based reconstruction, 40 iterations were performed, and geometry constraints were applied with 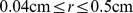, 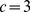, and targets centers inside the bounding box of the image object.

The fluorescence projection images in Fig. 8(b) show the high level of light scattering in turbid media. With the voxel-based method (R-ART), the dual inclusions were merged, and artifacts were present near the object boundary. In contrast, the two close targets were clearly separated by the proposed method with acceptable center deviations of 0.06 and 0.15 cm. The relative difference between the accumulated fluorescence intensities of the two targets was 8.4%, which may be partly caused by inevitable model error and the cross-talk between the two close inclusions. The actual free background was also accurately estimated. Overall, the results demonstrated that the proposed method has better resolution capability than traditional voxel-based reconstruction for practical applications.

A small animal experiment was performed to verify the feasibility of the proposed method for *in vivo* applications. This experiment was approved by the Science and Ethics Committee of the School of Biological Science and Medical Engineering in Beihang University, China. One nude mouse (5 weeks, 21 g) was anesthetized with pentobarbital and fixed on a glass plate holder, as shown in Fig. 8(a). A small fluorescence glass tube (0.3 cm diameter and 0.5 cm length, concentration of 4 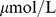) was embedded inside the nude mouse. The fluorescence and excitation projections were collected at a single projection angle. As shown in the first column of Fig. 8(c), the point light sources were scanned at 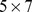 positions with steps of 0.3 cm. In the reconstruction, the mouse optical properties were assumed to be homogeneous (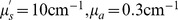) for simplicity. For voxel-based reconstruction, 30 R-ART iterations were performed with a relaxation parameter of 0.01. For shape-based reconstruction, 40 iterations were performed, and geometry constraints were applied with 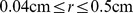, 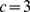, and target centers inside the bounding box of the image object. As shown in Fig. 8(c), the reconstructed fluorescence had a high value around the boundary and a widespread distribution inside the object. Compared to the actual single fluorescence inclusion, the reconstructed fluorescence was not acceptable. This was partly because of the limited projection angle, complex heterogeneous optical properties of the mouse, and the presence of an auto-fluorescent background. The proposed method, which benefited from the reduced number of unknowns, gave a better result. It recovered a single fluorescence inclusion and the background. This preliminary experiment demonstrated the feasibility of the proposed method for *in vivo* applications.

## Discussions and Conclusion

We proposed a shape-based reconstruction method for fluorescence molecular tomography that uses spherical harmonics parameterization. The inverse problem is formulated as a constrained nonlinear least-squares problem. To guarantee successful reconstruction and enhance robustness against initial conditions and noise, a two-step and modified block coordinate descent strategy was introduced to handle different shape parameters. Reasonable geometrical constraints are also enforced via the exterior penalty function method for further stability and accuracy. During the optimization, the objective function value and Jacobian matrix are calculated using the perturbation method, which is also greatly accelerated using GPU. The results of the numerical simulation and physical phantom and *in vivo* experiments all demonstrated the effectiveness of the proposed method.

Because of the incorporated shape priors and the resulting reduction in the dimensions of the inverse problem, the proposed method demonstrated better resolution capability than the conventional voxel-based method in the numerical and physical experiments. However, compared to voxel-based methods, the proposed method has the weakness of a relatively small application range. In application scenarios, the fluorescence distribution should be approximated as the sum of a small number of subdomains with piecewise constant intensities.

Although the number of unknowns is greatly reduced, attention should be paid to optimization techniques, as the shape-based reconstruction is still nonlinear and ill posed. If the difference in contributions to boundary measurements by the shape parameters is not considered and these parameters are simply recovered simultaneously, the reconstruction generally fails. In the proposed method, a two-step and modified block coordinate descent strategy is introduced. The optimization strategy stabilized the shape-based reconstruction against up to a 40% noise level. It also ensured the robustness of the proposed method against different initial values for noise and heterogeneity, even when the target number is not known *a priori*. In many cases, a fluorescent background is inevitable. As demonstrated in the numerical simulations, the proposed method worked well for low fluorescence contrasts down to 100∶20. This capacity was further verified in the *in vivo* experiment.

An intuitive explanation for the proposed optimization strategy is as follows. For a target, small deviations in its center position and expansion coefficients vary the boundary measurements for different methods. The center position deviation alters the distance from the target to different boundary sides; thus, it mainly changes the profiles of fluorescence projections. In contrast, the deviation of each expansion coefficient changes the target geometry and mainly changes the projection details. The difference in contributions requires the center position and expansion coefficients to be handled differently. In particular, the center position needs to be estimated first to approximate the coarse components of the fluorescence projections.

The proposed optimization strategy also has a mathematical explanation. For the Hessian matrixes of blocks 1 and 2 and all shape parameters, their condition numbers are different in orders of magnitude. For example, in the initial dual spheres case (Init 1 in Fig. 5), the condition numbers of the corresponding regularized Hessian matrixes (empirically selected regularization parameter of 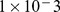) were 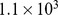, 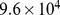, and 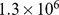, respectively. The high Hessian matrix condition number of block 2 means that the recovery of expansion coefficients is highly sensitive to noise and model error. Compared to block 2, the Hessian matrix condition number of block 1 was almost two orders of magnitude smaller, which means that the estimation of its variable elements is much less sensitive to error. This is why we use a two-step reconstruction scheme since the first step has the inherent advantage of much better stability. Compared to the two blocks, the Hessian matrix condition number for all shape parameters becomes even higher. Thus, when the shape parameters are recovered simultaneously in the second step, the estimation of the center positions is negatively affected by the expansion coefficients and becomes much more ill posed. Hence, the modified block coordinate descent strategy was adopted to alternately update blocks 1 and 2, which makes the second step more stable.

The convergence of the modified block coordinate descent optimization should be clarified since the standard coordinate descent method generally finds a local minimum. Although it is difficult to determine the convergence of the standard coordinate descent method [37] when the variables cannot be separated, modified block coordinate descent optimization can find a global minimum or near-global minimum in the presence of noise. The reasons are as follows. In the second step, the reconstruction has a relatively good initial estimate provided by the first step, especially for the center positions. Moreover, parameters with strong logical connections are put into the same block. In other words, the two blocks can be considered separable to some extent.

In the numerical simulation, the GPU accelerated the shape-based reconstruction about 30 times faster. The increased acceleration is important for the proposed method, as it guarantees the shape reconstruction time is only several (typically less than 3) minutes. By transforming the complex and frequently performed point-in-shape operation to a lookup table procedure, the GPU implementation becomes easier, and the computation speed becomes faster.

Apart from shape-based methods, total variation (TV) has attracted a great deal of attention in recent years since it can also strengthen the boundary edges between targets and background. Instead of directly incorporating shape priors, TV penalizes the intensity gradient information as the regularized term. Hence, it has the advantage of requiring fewer assumptions on the shape geometry and the weakness of not reducing the unknown dimensions. In recent years, TV has seen advances for FMT and demonstrated its superiority over traditional *L_2_* regularization in background-free cases [10], [11], [12]; future progress may demonstrate its effectiveness for low fluorescence contrast conditions. Since TV problem is highly nonlinear, its performance depends on the developed solution algorithm and selected regularization parameters. Hence, focus is presently on finding the optimal parameters or developing an automated parameter selection method. In comparison, the proposed method does not have the problem of determining regularization parameters, and selecting the geometry constraints for application is intuitive and simple. In general, the two techniques of shape-based reconstruction and TV are developing towards preserving the boundary edges for piece-constant fluorescence distributions. Each has its own strengths and weaknesses and thus needs further attention.

In this study, the normalized Born method [31] was used to reduce the negative effects of unknown heterogeneous optical properties. Shape parameterization can also be used to estimate the optical properties of different inner organs and helps better model the photon propagation inside small animals. Thus, the shape-based reconstruction quality can be further improved. In future work, we will focus on developing a full shape-based method to recover optical properties and successfully guide fluorescence distributions.
